# Acute Onset Rheumatoid Vasculitis With Polyarthritis and Erythema: A Case Report

**DOI:** 10.7759/cureus.48800

**Published:** 2023-11-14

**Authors:** Taiki Amao, Fusa Koda, Satoshi Ofuji, Chiaki Sano, Ryuichi Ohta

**Affiliations:** 1 Family Medicine, Shimane University Faculty of Medicine, Izumo, JPN; 2 Dermatology, Unnan City Hospital, Unnan, JPN; 3 Community Medicine Management, Shimane University Faculty of Medicine, Izumo, JPN; 4 Communiy Care, Unnan City Hospital, Unnan, JPN

**Keywords:** multidisciplinary approach, blood vessel inflammation, differential diagnosis, autoimmune processes, systemic joint pains, skin biopsy, erythema multiforme, skin manifestations, elderly onset, rheumatoid vasculitis

## Abstract

We present the case of a woman in her 70s who was diagnosed with rheumatoid vasculitis (RV) after initially presenting with systemic joint pain and erythema. RV, a rare complication of rheumatoid arthritis, involves inflammation of blood vessels, leading to various skin manifestations. The patient's complaints included fever, generalized joint pain, and skin manifestations that initially resembled erythema multiforme. However, a skin biopsy revealed vasculitis, which guided the RV diagnosis. Although rheumatoid arthritis primarily affects the joints, systemic implications such as RV can arise in rare cases. This case underscores the importance of a holistic and meticulous diagnostic approach, especially in older patients, as early detection and treatment are crucial for managing disease progression and associated complications. Collaborative care involving multidisciplinary teams is vital to achieving optimal outcomes in complex cases.

## Introduction

Rheumatoid vasculitis (RV) is a rare complication of rheumatoid arthritis, with 0.5% among all rheumatoid arthritis, in which inflammation affects the blood vessels [1]. It can lead to various skin manifestations, including petechiae, purpura, and skin ulcers in severe cases [2]. Though rheumatoid arthritis primarily targets the joints, systemic involvement can occasionally lead to conditions like RV [3]. Rheumatoid nodules, which are firm lumps that develop under the skin, typically near the joints, can also be associated with RV. They can vary in size and are often painless but can become tender or ulcerate [4]. Palindromic rheumatism, although mainly linked to rheumatoid arthritis, shares some clinical presentations with RV, characterized by sudden and recurrent episodes of painful swelling of the joints accompanied by reddish or purplish rashes around the affected areas [5]. In addition, RV develops in patients with advanced rheumatoid arthritis because of the deterioration of systemic inflammation. Thus, it is rare to diagnose patients with RV joint pain at the initial presentation of rheumatoid arthritis with joint pains. Here, we present the case of an older woman who initially presented with the chief complaint of polyarticular joint pain and erythema and was eventually diagnosed with rheumatoid vasculitis. This case report highlights the diverse presentations of RV in older individuals and offers a comprehensive diagnostic strategy for this condition.

## Case presentation

A woman in her 70s presented to our hospital with fever and generalized acute-onset pain as the main complaints. She had general malaise and arthralgia five days before admission and visited a rural community hospital's general medicine outpatient department two days before admission. She had a fever and a mildly swollen right knee. The patient was transferred to the emergency room because of a worsening respiratory condition. After symptomatic treatment, her vital signs stabilized, and she was temporarily sent home. The day before admission, she visited the orthopedic surgery department of her local doctor for a thorough examination of general joint pain, erythema, and palpable purpura in her upper and lower limbs with fever. On the day of admission, the patient visited our hospital's general medicine department. The medical history included hypertension, dyslipidemia, fatty liver, gallbladder polyps, and left pulmonary fractionation disease. Her drug history included the administration of enalapril (5 mg/day).

**Figure 1:**
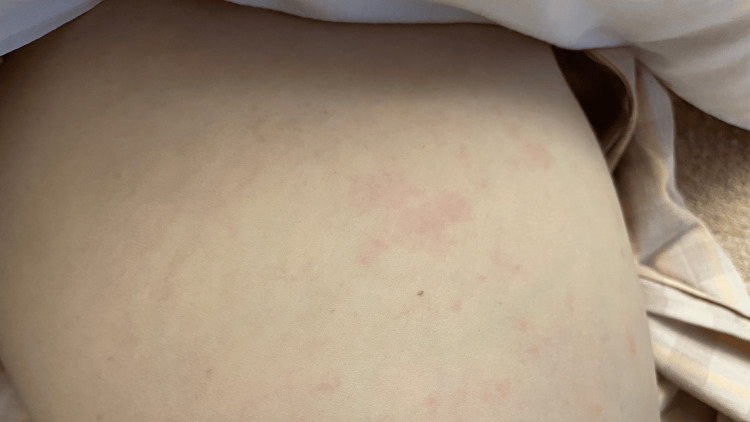
Erythema on the right thigh

Vital signs at the time of admission were a temperature of 36.5°C, blood pressure of 103/58 mmHg, pulse rate of 72 beats/min, respiratory rate of 15 breaths/min, and SpO₂ of 98%. Tenderness was observed in both shoulder, wrist, hands, and knee joints, and severe tenderness in both shoulders and thighs. There were no deformities of hands and feet without nodules. The whole part of the upper and lower extremities showed palpable oval and non-branching erythema and purpura without any itchiness (Figure 1).

The patient presented with fever and dyspnea at home and the previous medical institution, and the possibility of sepsis could not be ruled out. Blood samples revealed a hyperinflammatory state (Table 1).

**Table 1:** Initial laboratory test data of the patient eGFR - estimated glomerular filtration rate; CK - creatine kinase; CRP - C-reactive protein; Ig - immunoglobulin; C3 - complement component 3; C4 - component 4; KL-6 - Krebs von den Lungen-6; MPO-ANCA - myeloperoxidase-antineutrophil cytoplasmic antibody; PR3-ANCA - proteinase-3-antineutrophil cytoplasmic antibody; CCP - cyclic citrullinated peptide.

Parameter	Value	Reference
White blood cell count	6.40	3.5-9.1 × 10^3^/μL
Neutrophil differential count	85.7%	44.0-72.0%
Lymphocyte differential count	10.6%	18.0-59.0%
Monocyte differential count	2.7%	0.0-12.0%
Eosinophil differential count	0.7%	0.0-10.0%
Basophil differential count	0.3%	0.0–3.0%
Red blood cell count	3.89	3.76-5.50 × 10^6^/μL
Hemoglobin level	11.4	11.3-15.2 g/dL
Hematocrit volume	34.8%	33.4-44.9%
Mean corpuscular volume	89.5	79.0-100.0 fl
Platelet count	24.3	13.0-36.9 × 10^4^/μL
Erythrocyte sedimentation rate	48	3-15 mm
Total protein level	7.2	6.5-8.3 g/dL
Albumin level	3.6	3.8-5.3 g/dL
Total bilirubin level	0.4	0.2-1.2 mg/dL
Aspartate aminotransferase level	30	8-38 IU/L
Alanine aminotransferase level	20	4-43 IU/L
Alkaline phosphatase level	61	106-322 U/L
γ-Glutamyl transpeptidase level	240	<48 IU/L
Lactate dehydrogenase level	240	121-245 U/L
Blood urea nitrogen level	15.9	8-20 mg/dL
Creatinine level	0.57	0.40-1.10 mg/dL
eGFR	78.3	>60.0 mL/min/L
Serum Na level	138	135-150 mEq/L
Serum K level	3.7	3.5-5.3 mEq/L
Serum Cl level	103	98-110 mEq/L
Creatine kinase level	71	56-244 U/L
C-reactive protein level	19.81	<0.30 mg/dL
IgG level	1316	870-1700 mg/dL
Antinuclear antibody level	<40	<40
C3 level	142	86-160 mg/dL
C4 level	45	17-45 mg/mL
KL-6 level	421	105.3-401.2 U/mL
Rheumatoid factor level	39	<15 IU/mL
CCP antibody level	<0.6	<5 U/mL
MPO-ANCA	<1.0	<3.5 U/mL
PR3-ANCA	<1.0	<3.5 U/mL

Contrast-enhanced computed tomography (CT) of the thoracic and pelvic regions was performed to rule out deep site infection, abscess formation, and paraneoplastic syndrome causing systemic inflammatory arthritis, revealing no apparent abscesses or neoplastic lesions. Because of her age and acute presentation, we considered the possibility of sepsis due to intestinal migration from the intestinal tract and intra-abdominal infection and started ceftriaxone (2 g/day) infusion. Because the patient tested positive for rheumatoid factors and acute presentation, we initially diagnosed her with elderly onset rheumatoid arthritis based on ACR/EULAR 2010 score of eight. We started oral prednisolone (30 mg), as the joint X-ray of her hands and shoulders did not show any bone erosions and deformities. Considering the possibility of vasculitis, a biopsy of the erythema on the left lower leg was performed.

**Figure 2:**
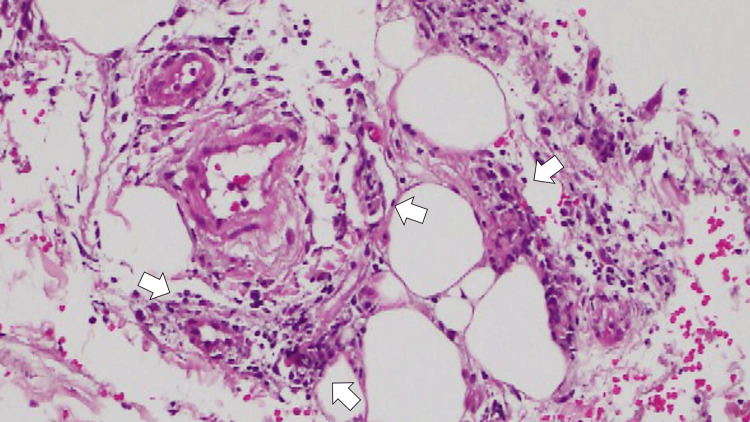
Pathological examination of the skin revealing stratified keratinization in the superficial layer of the skin and no significant findings in the epidermis (Hematoxylin and Eosin staining ×400) The dermis showed mild lymphocytic infiltration around blood vessels, and the subcutaneous fatty tissue showed degeneration, fibrosis, and macrophage infiltration, showing vasculitis (white arrows)

On the fifth day of hospitalization, after the above treatment, the patient's symptoms were relieved, she could walk independently, and her fever had resolved. On the same day, pathological examination revealed stratified keratinization in the superficial layer of the skin but no significant findings in the epidermis. The dermis showed mild lymphocytic infiltration around the blood vessels, and the subcutaneous fatty tissue showed degeneration, fibrosis, and macrophage infiltration. The vessels of the subcutaneous fatty tissue were infiltrated mainly by lymphocytes and neutrophils, indicating vasculitis (Figure 2).

Bacteremia was ruled out as blood cultures were negative on admission. Based on clinical and pathological presentations, the patient was diagnosed with rheumatoid vasculitis.

On day seven of hospitalization, all systemic skin rashes had resolved, and the antimicrobial therapy was discontinued after one week of treatment. The patient was well and was discharged. Subsequently, methotrexate (6 mg/week) was initiated on an outpatient basis, and the prednisolone dose was reduced.

## Discussion

We report a case of rheumatoid vasculitis with initial presentation of fever, skin rash, and polyarthritis in an older woman. Rheumatoid vasculitis, which is associated with a spectrum of rheumatoid diseases, presents with a specific and aggressive set of complications. A myriad of symptoms, including generalized skin rash, may manifest in cases of rheumatoid vasculitis with an elderly onset and acute progressive disease. This emphasizes the need for general practitioners to discern and rule out severe potential conditions.

Rheumatoid vasculitis is linked to autoimmune processes predominantly involving blood vessels as opposed to the joints seen in classic rheumatoid arthritis [3, 6]. Although skin rash is not typical in patients with rheumatoid arthritis, it is a more common manifestation when vasculitis is involved [7]. Vasculitis inflames the walls of blood vessels, and skin involvement is a known manifestation [8, 9]. Thus, when a skin rash is present in the context of rheumatoid disease, vasculitis should be considered in the differential diagnosis, rather than simply attributing it to rheumatoid arthritis.

Furthermore, skin rashes can indicate a severe form of rheumatoid disease or even an aggressive progression of vasculitis, which warrants immediate diagnosis and intervention [10]. Such conditions require early detection and treatment because of the risks associated with disease progression and complications [11]. Immediate skin biopsy can elucidate the exact nature and severity of the condition and guide appropriate therapeutic approaches. Hence, the presence of a skin rash should trigger an immediate skin biopsy to accurately discern its origin and implications.

While rheumatoid vasculitis primarily affects blood vessels, its systemic implications can be broad and severe. A skin rash can be a harbinger of escalated inflammation, offering prognostic insights [12, 13]. Rash due to sepsis can present a significant risk of mortality [14]. Moreover, patients with rheumatoid vasculitis inherently face heightened risks of complications, including further vascular processes [9, 15]. Given these clinical presentations, rheumatoid vasculitis should be differentiated from sepsis and other infections comprehensively.

Thus, a systematic approach is paramount for managing patients displaying signs of rheumatoid vasculitis with skin manifestations in community hospitals. Regular monitoring of vital signs can help assess circulatory and respiratory health, enabling early identification and intervention of potential deterioration. In this case, systematic approaches, including ruling out critical infections and prompt biopsies, could diagnose and treat rheumatoid vasculitis timely. Thus, comprehensive evaluations to ensure timely diagnosis and treatment are essential to effectively manage the risks associated with rheumatoid vasculitis and its complications.

General physicians should adopt a detailed systematic approach when presenting with suspected cases of rheumatoid vasculitis. Previous research has shown that these patients can have multiple coexisting conditions that require thorough diagnostic evaluation [16]. Collaboration with a multidisciplinary team driven by family physicians can optimize patient outcomes in rural community hospitals [17]. Therefore, a comprehensive, collaborative approach is necessary to ensure optimal patient care and outcomes.

## Conclusions

We encountered a woman in her 70s who was diagnosed with elderly onset rheumatoid arthritis complicated with rheumatoid vasculitis based on her primary symptoms of generalized joint pain and erythema. Rheumatoid vasculitis can present as skin manifestations. When a skin rash complicates rheumatoid vasculitis, it may indicate rapid inflammation and a rapid decline in the patient's overall health. Early skin biopsy is essential in cases of aggressive rheumatoid vasculitis, particularly in those presenting with skin complications.
